# Coccidioidomycosis Outbreaks, United States and Worldwide, 1940–2015

**DOI:** 10.3201/eid2403.170623

**Published:** 2018-03

**Authors:** Michael Freedman, Brendan R. Jackson, Orion McCotter, Kaitlin Benedict

**Affiliations:** Children’s Hospital of Pittsburgh, Pittsburgh, Pennsylvania, USA (M. Freedman);; Centers for Disease Control and Prevention, Atlanta, Georgia, USA (B.R. Jackson, O. McCotter, K. Benedict)

**Keywords:** Coccidioidomycosis, coccidioides, disease outbreaks, epidemiology, worldwide, Valley fever, fungi, United States

## Abstract

Coccidioidomycosis causes substantial illness and death in the United States each year. Although most cases are sporadic, outbreaks provide insight into the clinical and environmental features of coccidioidomycosis, high-risk activities, and the geographic range of *Coccidioides* fungi. We identified reports published in English of 47 coccidioidomycosis outbreaks worldwide that resulted in 1,464 cases during 1940–2015. Most (85%) outbreaks were associated with environmental exposures; the 2 largest outbreaks resulted from an earthquake and a large dust storm. More than one third of outbreaks occurred in areas where the fungus was not previously known to be endemic, and more than half of outbreaks involved occupational exposures. Coccidioidomycosis outbreaks can be difficult to detect and challenging to prevent given the unknown effectiveness of environmental control methods and personal protective equipment; therefore, increased awareness of coccidioidomycosis outbreaks is needed among public health professionals, healthcare providers, and the public.

Coccidioidomycosis, also known as Valley fever, is a disease caused by dimorphic fungi of the genus *Coccidioides* (*1*). The arthroconidia persist in soil, and environmental factors, including season, temperature, precipitation, and soil salinity, influence the organism’s life cycle (*2*–*4*). Coccidioidomycosis is known to be endemic to the southwestern United States, south-central Washington, northern Mexico, and parts of Central and South America (*1*). Infection occurs primarily by inhalation of environmental arthroconidia, and symptoms develop in ≈40% of infected persons typically within 1–3 weeks after exposure (*5*). However, determining the time and place of exposure is often difficult unless a notable exposure occurred or the illness is part of an outbreak. Symptomatic patients frequently have an influenza-like syndrome characterized by cough, shortness of breath, fever, and fatigue that is commonly diagnosed as community-acquired pneumonia. In ≈1% of infections, disseminated extrapulmonary disease (e.g., meningitis, osteomyelitis, or soft tissue and subcutaneous infections) develops (*1*,*6*,*7*). Risk factors for disseminated infection include immunocompromised status and African and Filipino ancestry (*1*). Serologic methods are the mainstay of diagnosis in coccidioidomycosis-endemic areas; diagnostic confirmation with identification of *Coccidioides* by culture or histopathology is less common (*8*). Treatment for mild coccidioidomycosis is often supportive; however, severe infections require treatment with antifungal agents (*1*).

Approximately 10,000 coccidioidomycosis cases are reported in the United States annually through reportable disease surveillance, and the substantial year-to-year variation that occurs reflects changing environmental conditions and testing practices (*9*). The disease is widely underdiagnosed, however, and these cases most likely represent a small fraction of the true number (*10*). Much remains unknown about its epidemiology. Although most reported cases represent sporadic infections (i.e., non–outbreak-associated), outbreak investigations can provide insight into coccidioidomycosis epidemiology because the exposure sites and circumstances are known, along with dates of exposure and illness. Data from outbreaks have informed much of the knowledge about the disease’s incubation period, environmental sources, geographic range, and high-risk activities, but coccidioidomycosis outbreaks have not been systematically studied. In 2016, we reviewed documented coccidioidomycosis outbreaks to identify common features and prevention opportunities.

## Methods

### Literature Review

We searched PubMed, Medline, Embase, Global Health, and Scopus without date or language restrictions for articles using combinations of the terms “coccidioides” or “coccidioidomycosis” and “epidemic,” “outbreak,” or “cluster.” We also searched the digital archive of scientific literature produced by the Centers for Disease Control and Prevention (Atlanta, Georgia, USA) Stacks database (http://stacks.cdc.gov) for any reports with the words “coccidioidomycosis” or “coccidioides” published in Morbidity and Mortality Weekly Report before 1981. We reviewed proceedings from annual Coccidioidomycosis Study Group meetings held during 1980–2015 for abstracts describing possible outbreaks (*11*) and searched National Institute for Occupational Safety and Health Hazard Evaluation reports using the terms “coccidioidomycosis” or “coccidioides” (*12*). We reviewed all references pertaining to outbreaks in all relevant articles and reviewed any references cited within the initial article if they alluded to additional outbreaks or additional information for a known outbreak. We included in the analysis English-language articles published during January 1940–August 2016. We abstracted clinical and epidemiologic data of interest from reports of outbreaks that met these inclusion criteria.

### Definitions

We defined an outbreak as >2 human coccidioidomycosis cases linked to a common source, event, or activity in space and time. For an outbreak to be included, laboratory evidence of coccidioidomycosis was required for >1 case. However, to capture older literature describing outbreaks most likely attributable to coccidioidomycosis, we also included outbreaks if cases had a compatible clinical syndrome and unambiguous diagnostic terms, such as “coccidioidomycosis” or “primary pulmonary coccidioidomycosis,” and were described in a report that also mentioned >1 patient with 1) laboratory evidence of coccidioidomycosis, 2) hospitalization, or 3) identification of *Coccidioides* from the environmental source.

We reviewed laboratory and radiologic evidence of infection. Specific laboratory tests documented as positive were serology (including immunodiffusion, precipitins, complement fixation positive in any titer, and enzyme immunoassay); culture or direct visualization (i.e., smear or microscopy) of *Coccidioides* in any body fluid; PCR; and coccidioidin skin test (any reaction at any dilution). We considered infiltrates or abnormalities on chest radiography radiographic evidence of infection, whereas we defined radiograph findings reported as equivocal or nonspecific as equivocal. Symptomatic patients were those with clinical symptoms consistent with coccidioidomycosis, regardless of diagnostic studies.

We classified outbreaks as environmental or nonenvironmental (i.e., resulting from healthcare or laboratory exposure) in origin. We further subclassified environmental outbreaks by whether a probable exposure source (e.g., soil disruption, dust storm) was identified. Environmental outbreaks without a probable exposure source were limited to those clearly bound by beginning and end time points; we excluded generalized increases in incidence among specific populations. We further characterized outbreaks by their association with military activity, incarceration, residential areas, laboratory activities, archaeology and field studies, or travel. Incarceration included military and civilian incarceration, as well as any group of persons detained against their will (e.g., in Japanese internment camps). Outbreaks were classified as residential if the report described the outbreak at or near a residential area, including the residences of the persons exposed. Outbreaks were determined to be travel-associated if travel was explicitly mentioned, and all military outbreaks were also considered to be travel-associated.

Outbreak location was defined as the geographic location of the probable exposure source or the patients’ location when no source was identifiable. Occupational exposures were those clearly related to employment, as well as activities associated with archaeology, field studies, and military imprisonment. We also assessed reports for statements about whether outbreaks occurred in areas where, according to the original authors, coccidioidomycosis was not previously known to be endemic.

We determined epidemiologic metrics on the basis of the original authors’ definitions and defined the number of persons possibly exposed as those exposed to the probable source, if present. We also recorded the number of cases resulting in hospitalization, dissemination, meningitis, or death in outbreak reports that described any of these clinical outcomes. We considered patients with meningitis or sepsis to have disseminated disease. We documented use of antifungal drugs when available and recorded or estimated minimum, maximum, median, and mean symptom durations from figures and individual case reports when available. We further documented data pertaining to incubation periods for outbreaks that had total exposure periods (i.e., date of last exposure minus date of first exposure) lasting <2 weeks.

We directly documented all data in our analysis from the literature or estimated data using conservative assumptions (e.g., majority of cases was defined as 50% + 1). In the absence of explicit data or the above conservative estimates, we noted information as unknown and omitted it from our analysis.

## Results

A total of 47 coccidioidomycosis outbreaks met our study criteria (Technical Appendix). These outbreaks involved 1,464 cases (1,451 symptomatic and 13 asymptomatic) (Table 1). An additional 128 symptomatic persons were reportedly affected by these outbreaks, but their illnesses did not meet the original authors’ case criteria. Outbreaks ranged in size from 2 to 379 cases (mean 31, median 10). Five (11%) outbreaks had 2 cases each, 8 (17%) had 3–5 cases, 8 (17%) had 6–9 cases, 12 (26%) had 10–14 cases, 9 (19%) had 15–99 cases, and 5 (11%) had >100 cases.

**Table 1:** Characteristics of published coccidioidomycosis outbreaks and outbreak-associated cases, United States and worldwide, 1940–2015

Outbreak characteristic	Outbreaks, no. (%), N = 47	Cases, N = 1,464	
Total no. (%)	Median (min–max)*
Environmental†	40 (85)	1,425 (97)	10 (2–379)
Probable source of exposure reported‡	35 (88)	1,218 (85)	10 (2–379)
Associated with large natural phenomena	2 (4)	582 (40)	291 (203–379)
Revealed new, or confirmed suspected, endemic area§	16 (43)		
Occupational	25 (53)	616 (42)	10 (2–150)
Military	11 (23)	442 (30)	14 (8–150)
Archaeology/field studies	7 (15)	82 (6)	10 (5–27)
Laboratory	4 (9)	28 (2)	5.5 (2–5)
Construction¶	7 (15)	247 (17)	21 (8–119)
Other activity			
Armadillo hunting in northern Brazil	5 (11)	14 (1)	3 (2–4)
Native American site disruption	2 (4)	6 (<1)	3 (2–4)
Location			
Travel-associated	21 (45)	566 (39)	12 (5–150)
Residential	8 (17)	625 (43)	9 (2–379)
Incarceration	5 (11)	316 (22)	30 (7–150)

*Max, maximum; min, minimum. †That is, not laboratory- or healthcare-associated. ‡Of 40 environmental outbreaks with 1,425 outbreak-associated cases. §Of 37 environmental outbreaks for which this information was available. ¶Three outbreaks associated with construction were not considered occupational outbreaks, including 2 associated with construction by volunteers in Mexico and 1 associated with construction adjacent to a prison in California’s Central Valley.

**Figure 1:**
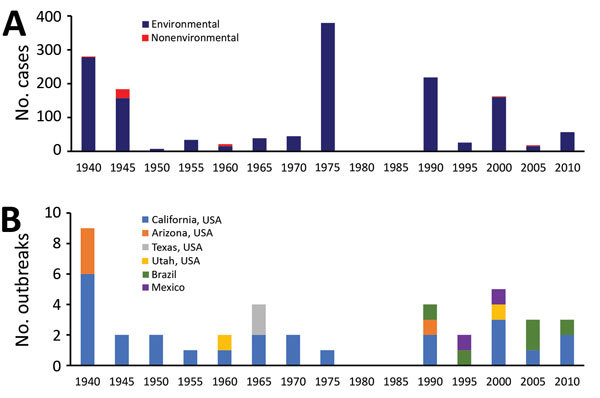
Coccidioidomycosis cases, United States and worldwide, 1940–2015. A) Outbreak-related cases, by onset year (in 5-year periods) and environmental association (N = 1,464 cases). B) Environment-associated outbreaks, by onset year and outbreak location (N = 40 outbreaks).

More than 60% of cases occurred during 1940–1949 (32%) and 1970–1979 (29%) (Figure 1, panel A). Among the 33 (70%) environment-associated outbreaks in the Northern Hemisphere for which onset month or season were reported, 10 (30%) started in summer (June–August).

Thirty-one (66%) outbreaks reported total numbers of persons possibly exposed, ranging from 2 to 676,667 (median 27). Clinical attack rates ranged from 0.03% to 100% (mean 44%, median 43%).

### Exposure Characteristics

**Figure 2:**
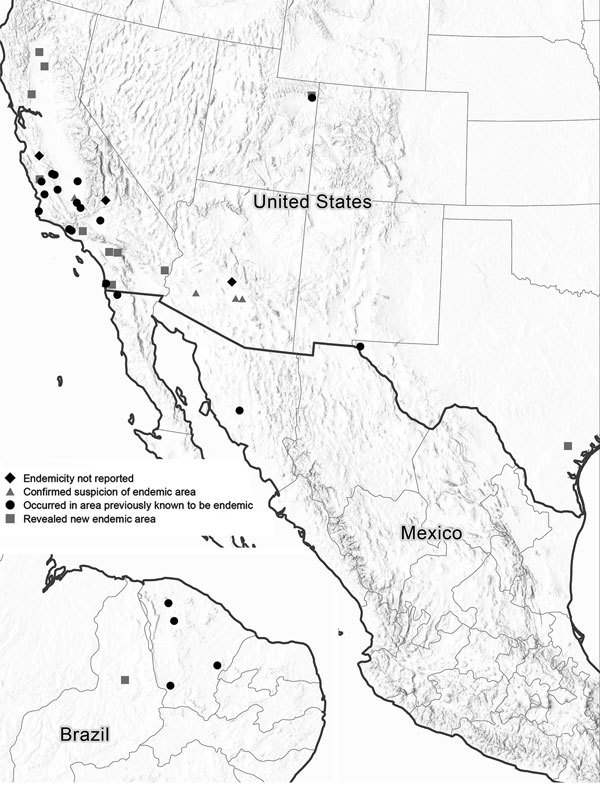
Locations of environment-associated coccidioidomycosis outbreaks, by state or territory and whether the outbreak revealed new or confirmed suspected endemicity (n = 40), United States, Mexico, and Brazil, 1940–2015.

Forty (85%) outbreaks were associated with environmental exposure, 33 (83%) of which occurred in the United States. Among the US environment-associated outbreaks, 25 (76%) occurred in California, 4 (12%) in Arizona, and 2 (6%) each in Utah and Texas (Figure 1, panel B). Of the remaining 7 (18%) environment-associated outbreaks, 5 (71%) occurred in Brazil and 2 (29%) in Mexico (Figure 2). Thirty-five (88%) environment-associated outbreaks involved a documented probable exposure source.

We found 7 (15%) nonenvironmental outbreaks. Four of these were related to laboratory exposures, 2 involved transmission through organ transplantation, and 1 was a nosocomial outbreak.

Two (4%) outbreaks comprising 582 (40%) cases resulted from separate natural phenomena in California: the Northridge earthquake in 1994 in Ventura County and the “Tempest from Tehachapi” dust storm in 1977. Twenty-one (45%) outbreaks comprising 566 (39%) cases were travel-associated. There were 316 (22%) cases in the 5 (11%) outbreaks among incarcerated populations, including 160 cases from 2 outbreaks in prisoner-of-war camps, 30 cases from an outbreak in a Japanese internment camp, 119 cases from an outbreak in a civilian prison, and 7 cases from an outbreak in a juvenile work camp.

Thirty-seven (79%) outbreaks reported whether the given outbreak revealed previously unknown information about endemicity. Of these, 12 (32%) revealed a new area (in Arizona, California, Texas, Utah, and Brazil) to which coccidioidomycosis was endemic, and 4 (11%) confirmed the endemicity of a suspected endemic area (in Arizona and California).

### Occupational Exposures

Twenty-five (53%) outbreaks were associated with occupational exposures. Eleven (23%) outbreaks were associated with the military. Seven (15%) outbreaks were associated with construction, and another 7 (15%) were associated with archaeology or other field studies. Laboratory activities were associated with 4 (9%) outbreaks.

### Incubation Period and Symptom Duration

Fifteen (32%) outbreaks included incubation period data meeting predefined inclusion criteria. Fourteen of these outbreaks stated lower and upper incubation period limits; the lower limit ranged from 1 to 14 days (mean 7, median 8) and the upper limit from 4 to 70 days (mean 21, median 17). The median incubation period, reported for 12 outbreaks, ranged from 4 to 31 days (mean 12, median 13), and the mean incubation period, reported for 11 outbreaks, ranged from 4 to 33 days (mean 14, median 12).

Of the 13 (28%) outbreak reports that included symptom duration, the lower limit ranged from 1 day to 17 weeks (mean 3.4 weeks, median 2 weeks), and the upper limit ranged from 3 to 52 weeks (mean 16.2, median 10). The median symptom duration ranged from 1 to 18 weeks (mean 7.1, median 4.5), and the mean symptom duration ranged from 3 to 19.6 weeks (mean 9.3, median 6.3).

### Laboratory and Radiologic Evidence

Thirty-nine (83%) outbreaks had cases confirmed by laboratory evidence. Radiographic evidence was reported for 153 (10%) cases in 22 (47%) outbreaks (Table 2). Among the 1,464 cases, at least 1,001 (68%) reported diagnostic evidence of coccidioidomycosis, including 334 with positive serologic results, 30 with positive culture or histopathologic visualization results, 378 with positive skin test results, and 153 with positive chest radiograph results.

**Table 2:** Characteristics of patients with coccidioidomycosis outbreak–associated cases, United States and worldwide, 1940–2015

Patient characteristic	Time period, no. (%) cases			
1940–1959	1960–1979	1980–2015	Total 1940–2015
Presentation and diagnosis				
Total	503 (100)	482 (100)	479 (100)	1,464 (100)
Symptomatic	493 (98)	481 (100)	477 (100)	1,451 (99)
Any positive studies	290 (58)	466 (97)	242 (51)	998 (68)
Positive serology	156 (31)	68 (14)	110 (23)	334 (23)
Positive skin test result	270 (54)	82 (17)	26 (5)	378 (26)
Positive culture	15 (3)	2 (<1)	13 (3)	30 (2)
Positive chest radiograph	50 (10)	67 (14)	36 (8)	153 (10)
Clinical outcome				
Total*	475 (100)	472 (100)	320 (100)	1,267 (100)
Hospitalized	430 (91)	20 (4)	94 (29)	544 (43)
Dissemination	7 (1)	16 (3)	9 (3)	32 (3)
Meningitis	3 (1)	15 (3)	2 (1)	20 (2)
Deaths	5 (1)	8 (2)	5 (2)	18 (1)
Treated with antifungal drugs†	0	5 (1)	123 (44)	128 (18)

*Cases among outbreaks reporting health outcomes. †Of 717 cases among 20 outbreaks documenting antifungal drug treatment.

### Treatment and Outcomes

Health outcome data were available for 35 (74%) outbreaks in which 544 (43%) patients were hospitalized (Table 2). Thirty-two (3%) patients had disseminated coccidioidomycosis and 20 (2%) had meningitis; 18 (1%) died. Of the 20 (43%) outbreaks that described antifungal treatment, 128 (18%) patients in 13 outbreaks were treated with antifungal drugs, including amphotericin B, fluconazole, and other azole antifungals. 

## Discussion

We reviewed 47 coccidioidomycosis outbreaks comprising 1,464 cases, for an average of 19 outbreak-related cases annually during 1940–2015. Although reported outbreak-related cases are relatively uncommon compared with the thousands of annual reported cases in the United States alone, coccidioidomycosis outbreaks have helped inform our understanding of geographic risk, high-risk populations and activities, and clinical features of the infection.

Outbreaks have further defined the geographic distribution of *Coccidioides*, initially identified in part by large-scale coccidioidin skin test surveys (*13*). In fact, 7 of the first 9 environmental coccidioidomycosis outbreaks in the early 1940s either confirmed suspicion of or revealed previously unknown areas in southern California and western Arizona to which the fungus is endemic. Over time, outbreak investigations uncovered additional coccidioidomycosis-endemic areas throughout California’s Central Valley, Texas, Utah, and areas of Brazil. Of outbreak reports that described endemicity, nearly half (43%) involved outbreaks that occurred in locations where the infection was not known to be endemic. Outbreak data also suggest that some geographic regions seem particularly well suited for *Coccidioides* growth and human exposure. For example, the arid hills bordering the southwestern portion of California’s Central Valley were the setting for 5 outbreaks within 150 miles of each other.

Although outbreaks can help identify areas with geographic risk for coccidioidomycosis, few outbreaks have been reported from some regions to which coccidioidomycosis is known to be highly endemic. Specifically, only 4 outbreaks were reported from Arizona (3 of which occurred in the early 1940s), even though several Arizona counties report some of the highest coccidioidomycosis incidence rates nationwide. In contrast, 25 outbreaks occurred in California, a state with localized areas of similarly high incidence. A possible explanation for this discrepancy is that most of Arizona’s population resides in areas where coccidioidomycosis is highly endemic, and exposure probably is common during daily activities, resulting in many sporadic cases and making outbreak detection challenging in this setting of high baseline incidence. In contrast, much of California’s population resides outside of its most highly coccidioidomycosis-endemic areas, possibly enabling easier outbreak detection against lower baseline rates. Another possible contributing explanation for this discrepancy is that cases of coccidioidomycosis have been determined to be a compensable work-related condition in California, whereas cases in Arizona have not, which might influence outbreak recognition (*14*). However, in a recent work-related outbreak in California, public health investigators initially identified few cases by reviewing workers’ compensation claims, highlighting the need for data integration from multiple sources to identify and describe some outbreaks (*15*). Systematic collection of information on occupation, industry, and workplace as part of coccidioidomycosis surveillance might facilitate identifying future workplace-associated outbreaks. Outbreak detection is challenging in general because Arizona and many counties in California currently use laboratory-based coccidioidomycosis surveillance and patients are not routinely interviewed to detect possible common exposures.

In addition to the challenges associated with outbreak detection, defining whether a cluster of coccidioidomycosis cases or a period of elevated incidence truly represents an outbreak can be difficult, particularly when a specific exposure source is not apparent. Coccidioidomycosis incidence fluctuates with season and weather (*16*), and increased reports resulting from seasonal sporadic infection could be interpreted as an outbreak. For this review, we excluded several reports noting increased incidence over a prolonged period, despite specific identification with the terms “outbreak” or “epidemic” (*16*–*18*), to focus on outbreaks with more clearly defined sources. Outbreaks without a probable exposure source included in this review had a clear association among a group of persons, frequently those who traveled from areas to which coccidioidomycosis is not known to be endemic. Periods of widespread, elevated coccidioidomycosis incidence associated with seasonal or weather changes contrast with outbreaks clearly resulting from defined natural phenomena. The 2 largest outbreaks in this review, resulting from the 1994 Northridge Earthquake (*19*) and a 1977 dust storm originating near the Tehachapi mountains in Southern California (*20*), together comprised 40% of all outbreak-related cases, illustrating the potential for these events to affect many persons across large geographic areas.

This review highlights the well-recognized risk for coccidioidomycosis among several specific populations, including military personnel, incarcerated persons, and outdoor workers. Many of the earliest outbreaks occurred among service members stationed in semiarid desert areas of Arizona and California during and shortly after World War II (*21*,*22*). These early outbreaks were studied extensively by C.E. Smith, who advocated for dust control measures, such as planting grass, paving, and applying oil to soil in athletic areas, to reduce risk for infection (*21*–*23*). Three exclusively military-associated outbreaks have been described since then (1958, 1992, and 2001), resulting in many fewer cases than those in the early 1940s (51 vs. 391 cases). Coccidioidomycosis continues to be reported among service members (*24*,*25*), most likely as a consequence of immune-naive persons entering areas to which it is endemic, although evidently not to the same magnitude as during the World War II era.

This review also highlights the ongoing challenge of coccidioidomycosis in California prisons. The 5 outbreaks among incarcerated persons were similar to those among the military in that affected persons often did not have prior exposure to coccidioidomycosis-endemic areas (*22*,*26*–*29*). In an effort to minimize illness, inmates who are immunosuppressed, are African-American or Filipino, or have diabetes mellitus are no longer housed in several prisons in California’s Central Valley (*30*). Additionally, inmates are offered coccidioidal skin testing to further reduce the risk for contracting coccidioidomycosis in prisons located in areas to which it is highly endemic (*31*).

More than half of the coccidioidomycosis outbreaks we reviewed were associated with occupational exposure, often related to soil-disrupting activities. The association between coccidioidomycosis and these activities, specifically construction, is often described, although evidence from a nonoutbreak setting in Arizona suggests that exposure working near a construction site does not always appear to be associated with increased risk for coccidioidomycosis (*32*). In general, workers who disrupt soil in areas to which the fungus is endemic are believed to be at higher risk than the general population. Recommendations from the California Department of Public Health (*33*) and the United States Geological Survey (*34*) to prevent work-related coccidioidomycosis focus on strategies such as education about coccidioidomycosis for workers and supervisors, dust-control methods such as wetting soil before disrupting it, cleaning potentially contaminated materials to prevent *Coccidioides* from being transported away from the worksite, and using respiratory protection; however, the efficacy of these interventions is difficult to measure. Furthermore, these types of control measures can be difficult to implement or enforce. For example, during a 2011 outbreak among construction workers building a solar power farm in San Luis Obispo County, California, 88% of interviewed patients reported receiving safety training on Valley fever, but their descriptions of the training varied widely (*15*). Clearly, further research on preventing work-related coccidioidomycosis is needed.

Nonenvironmental coccidioidomycosis outbreaks were uncommon, and most were caused by laboratory exposure. *Coccidioides* cultures can be highly infectious; 1 incident caused 15 coccidioidomycosis cases, affecting persons in several rooms on the same floor (*35*). No laboratory-associated outbreaks have been published since 1949, consistent with notably improved prevention and laboratory safety measures (*36*).

With the advent of transplant medicine, at least 2 coccidioidomycosis outbreaks have been associated with organ transplantation, 1 of which was confirmed by whole-genome sequencing of isolates from 3 organ recipients (*37*). Coccidioidomycosis poses a serious risk for transplant recipients, and further studies are needed to determine whether serologic screening on donors from coccidioidomycosis-endemic areas minimizes transmission (*38*).

Clinical features of coccidioidomycosis cases in this review were similar to those in previous studies. The median shortest incubation period was 8 days, median longest 17 days, and overall median 13 days, consistent with the commonly reported incubation period range of 1 to 3 weeks (*39*). The median shortest symptom duration was 2 weeks, the median longest was 10 weeks, and the overall median was 4.5 weeks. By comparison, among patients with reported coccidioidomycosis in Arizona, median symptom duration was ≈6 weeks among patients who had recovered at the time of the interview and ≈22 weeks among patients who had not recovered (*40*). A possible explanation for this difference is that cases reported to public health might be more severe than those that go unreported, whereas cases detected as part of outbreaks might reflect a wider spectrum of illness; in addition, many cases detected by surveillance occur in older adults who might have comorbidities and slower recovery times, whereas many outbreak-associated cases occurred in occupational settings and most likely affected younger adults. However, the median clinical attack rate (43%) and proportions of patients who were hospitalized (43%), had disseminated disease (3%), and died (1%) were generally similar to those described elsewhere (*5*–*7*,*40*,*41*), suggesting that other factors might explain the differences in symptom duration observed. Notably, hospitalization varied substantially by time period; 91% of patients during 1940–1959 were hospitalized but only 29% during 1980–2015. This trend follows an increase in antifungal drug use from 0% to 44% during the same period. These patterns probably reflect changes in medical practice.

This review is limited by the fact that many outbreaks probably are not recognized, not reported to public health, not investigated, or not published, although we also attempted to capture reports from the gray literature. Other limitations include the heterogeneity of available data and our restriction to English-language reports.

Nevertheless, outbreaks are a key data source regarding modes and locations of exposure. Thus, increased attention to outbreak identification and tracking is worthwhile given the continued population growth in coccidioidomycosis-endemic areas, increased settlement at the wildland–urban interface, and the incompletely understood effects of intensifying climate change on *Coccidioides*. Monitoring outbreaks could be critical in identifying new areas of endemicity and high-risk activities. Increased awareness of coccidioidomycosis among employers of persons in potentially high-risk occupations, the public, and healthcare providers is needed to reduce both the risk and severity of future outbreaks.

Technical Appendix Chronologic list of coccidioidomycosis outbreaks, United States and worldwide, 1940–2015.
