# Supplementary material for: Coccidioidomycosis Outbreaks, United States and Worldwide, 1940–2015
Source: Emerg Infect Dis. 2018 Mar;24(3):417–24. doi: 10.3201/eid2403.170623 (PMC5823332; doi:10.3201/eid2403.170623)
Supplement: Technical Appendix — Chronologic list of coccidioidomycosis outbreaks, United States and worldwide, 1940–2015. [file 17-0623-Techapp-s1.pdf]

Article DOI: https://doi.org/10.3201/eid2403.170623

Coccidioidomycosis Outbreaks, United States and Worldwide, 1940–2015

Technical Appendix

Technical Appendix Table. Chronologic list of coccidioidomycosis outbreaks, United States and worldwide, 1940–2015

Author, publication year	Outbreak year	No. cases
Davis et al, 1942 (*1*)	1940	7
Shelton, 1942 (*2*)	1941	14
Goldstein et al, 1943 (*3*)	1942	75
Smith, 1958 (*4*)	1942	30
Willett et al, 1945 (*5*)	1943	12
Willett et al, 1945 (*5*)	1943	100
Goldstein et al, 1944 (*6*)	1943	10
Smith, 1958 (*7*)	1943	20
Smith, 1958 (*7*)	1943	10
Hanel et al, 1967 (*8*)	1944	2
Smith, 1958 (*7*)	1945	150
Hanel et al, 1967 (*8*)	1946	4
Smith et al, 1948 (*4*)	1946	7
Kritzer et al, 1950 (*9*)	1948	7
Hanel et al, 1967 (*8*)	1949	15
Walch et al, 1961 (*10*)	1950	2
Plunket et al, 1957 (*11*)	1954	5
Joffe, 1960 (*12*)	1958	33
Eckman et al, 1964 (*13*)	1961	6
Winn et al, 1963 (*14*)	1961	10
Petersen et al, 2004 (*15*)	1964	5
Roberts et al, 1967 (*16*)	1965	10
Teel et al, 1970 (*17*)	1966	9
Ramras et al, 1970 (*18*)	1966	8
Loofburrow et al, 1969 (*19*)	1968	11
Werner et al, 1972 (*20*)	1970	27
Werner et al, 1973 (*21*)	1972	17
Pappagianis et al, 1978 (*22*)	1977	379
Wanke et al, 1999 (*23*)	1991	3
Thomas et al, 1993 (*24*)	1992	4
Standaert et al, 1995 (*25*)	1992	8
Schneider et al, 1997 (*26*)	1994	203
de Aguiar Cordeiro et al, 2010 (*27*)	1995	4
Cairns et al, 2000 (*28*)	1996	21
Centers for Disease Control and Prevention, 2000 (*29*)	2000	8
Wright et al, 2003 (*30*)	2000	2
Clark et al, 2002 (*31*)	2001	13
Crum et al, 2002 (*32*)	2001	10
Petersen et al, 2004 (*15*)	2001	10
Pappagianis et al, 2004 (*33*)	2002	119
de Aguiar Cordeiro et al, 2010 (*27*)	2006	3
de Aguiar Cordeiro et al, 2010 (*27*)	2007	2
Cummings et al, 2010 (*34*)	2007	10
Engelthaler et al, 2011 (*35*)	2009	3
Brillhante et al, 2012 (*36*)	2010	2
Wilken et al, 2015 (*37*)	2011	44
Wilken et al, 2014 (*38*)	2012	10

References

1. Davis BL Jr, Smith RT, Smith CE. An epidemic of coccidioidal infection (coccidioidomycosis). J Am Med Assoc. 1942;118:1182–6. http://dx.doi.org/10.1001/jama.1942.02830140012004

2. Shelton RM. A survey of coccidioidomycosis at Camp Roberts, California. J Am Med Assoc. 1942;118:1186–90. http://dx.doi.org/10.1001/jama.1942.02830140016005

3. Goldstein D, Louie S. Primary pulmonary coccidioidomycosis. Report of an epidemic of seventy-five cases. War Med (Chic). 1943;4:299–317.

4. Smith DT, Harrell ER Jr. Fatal coccidioidomycosis; a case of a laboratory infection. Am Rev Tuberc. 1948;57:368–74. PubMed

5. Willett F, Weiss A. Coccidioidomycosis in southern California: report of a new endemic area with a review of 100 cases. Ann Intern Med. 1945;23:349–75. http://dx.doi.org/10.7326/0003-4819-23-3-349

6. Goldstein DM, McDonald JB. Primary pulmonary coccidioidomycosis: follow-up of 75 cases, with 10 more cases from a new endemic area. J Am Med Assoc. 1944;124:557–61. http://dx.doi.org/10.1001/jama.1944.02850090013004

7. Smith CE. Coccidioidomycosis: historical note. In: Coates JB, Hoff EC, Hoff MA, editors. Preventive medicine in World War II, Volume IV, communicable diseases, transmitted chiefly through respiratory and alimentary tracts. Washington (DC): Office of the Surgeon General, Medical Department, United States Army; 1958. p. 285–316.

8. Hanel Jr E, Kruse RH. Miscellaneous publication 28: laboratory-acquired mycoses. Fort Detrick, Frederick (MD): Industrial Health and Safety Office, Research and Radiological Division, Department of the Army; 1967.15. Roberts PL, Lisciandro RC. A community epidemic of coccidioidomycosis. Am Rev Respir Dis. 1967;96:766–72. PubMed

9. Kritzer MD, Biddle M, Kessel JF. An outbreak of primary pulmonary coccidioidomycosis in Los Angeles County, California. Ann Intern Med. 1950;33:960–90. PubMed http://dx.doi.org/10.7326/0003-4819-33-4-960

10. Walch HA, Pribnow JF, Wyborney VJ, Walch RK. Coccidioidomycosis in San Diego County and the involvement of transported topsoil in certain cases. Am Rev Respir Dis. 1961;84:359–63. PubMed

11. Plunket OA, Swatek FE. Ecological studies of *Coccidioides immitis*. Proceedings of Symposium on Coccidioidomycosis; 1957: U.S. Public Health Bulletin no. 575; 1957. p. 158–60.

12. Joffe B. An epidemic of coccidioidomycosis probably related to soil. N Engl J Med. 1960;262:720–2. PubMed http://dx.doi.org/10.1056/NEJM196004072621408

13. Eckmann BH, Schaefer GL, Huppert M. Bedside interhuman transmission of coccidioidomycosis via growth on fomites. An epidemic involving six persons. Am Rev Respir Dis. 1964;89:175–85. PubMed

14. Winn W, Levine H, Broderick J, Crane R. A localized epidemic of coccidioidal infection: primary coccidioidomycosis occurring in a group of ten children infected in a backyard playground in the San Joaquin Valley of California. N Engl J Med. 1963;268:867–70. http://dx.doi.org/10.1056/NEJM196304182681604

15. Petersen LR, Marshall SL, Barton C, Hajjeh RA, Lindsley MD, Warnock DW, et al. Coccidioidomycosis among workers at an archeological site, northeastern Utah. Emerg Infect Dis. 2004;10:637–42. PubMed http://dx.doi.org/10.3201/eid1004.030446

16. Roberts PL, Lisciandro RC. A community epidemic of coccidioidomycosis. Am Rev Respir Dis. 1967;96:766–72. PubMed

17. Teel KW, Yow MD, Williams TW Jr. A localized outbreak of coccidioidomycosis in southern Texas. J Pediatr. 1970;77:65–73. PubMed http://dx.doi.org/10.1016/S0022-3476(70)80046-4

18. Ramras DG, Walch HA, Murray JP, Davidson BH. An epidemic of coccidioidomycosis in the Pacific Beach area of San Diego. Am Rev Respir Dis. 1970;101:975–8. PubMed

19. Loofbourow JC, Pappagianis D, Cooper TY. Endemic coccidioidomycosis in northern California. An outbreak in the Capay Valley of Yolo County. Calif Med. 1969;111:5–9. PubMed

20. Werner SB, Pappagianis D, Heindl I, Mickel A. An epidemic of coccidioidomycosis among archeology students in northern California. N Engl J Med. 1972;286:507–12. PubMed http://dx.doi.org/10.1056/NEJM197203092861003

21. Werner SB, Pappagianis D. Coccidioidomycosis in northern California. An outbreak among archeology students near Red Bluff. Calif Med. 1973;119:16–20. PubMed

22. Pappagianis D, Einstein H. Tempest from Tehachapi takes toll or *Coccidioides* conveyed aloft and afar. West J Med. 1978;129:527–30. PubMed

23. Wanke B, dos Santos Lazera M, Monteiro PCF, Lima FC, Leal MJS, Ferreira Filho PL, et al. Investigation of an outbreak of endemic coccidioidomycosis in Brazil's northeastern state of Piauí with a review of the occurrence and distribution of *Coccidioides immitis* in three other Brazilian states. Mycopathologia. 1999;148:57–67. PubMed http://dx.doi.org/10.1023/A:1007183022761

24. Thomas A, Sarosi G, Smith D. Coccidioidomycosis outbreak on the Salt River Indian Reservation. In: Proceedings of the Thirty Seventh Annual Coccidioidomycosis Study Group Meeting; 1993 Apr 3; Tucson (AZ). Tucson (AZ); Valley Fever Center for Excellence; 1993. p. 8.

25. Standaert SM, Schaffner W, Galgiani JN, Pinner RW, Kaufman L, Durry E, et al. Coccidioidomycosis among visitors to a *Coccidioides immitis*–endemic area: an outbreak in a military reserve unit. J Infect Dis. 1995;171:1672–5. PubMed http://dx.doi.org/10.1093/infdis/171.6.1672

26. Schneider E, Hajjeh RA, Spiegel RA, Jibson RW, Harp EL, Marshall GA, et al. A coccidioidomycosis outbreak following the Northridge, Calif, earthquake. JAMA. 1997;277:904–8. PubMed http://dx.doi.org/10.1001/jama.1997.03540350054033

27. de Aguiar Cordeiro RA, Brilhante RSN, Rocha MFG, Bandeira SP, Fechine MAB, de Camargo ZP, et al. Twelve years of coccidioidomycosis in Ceará State, northeast Brazil: epidemiologic and diagnostic aspects. Diagn Microbiol Infect Dis. 2010;66:65–72. PubMed http://dx.doi.org/10.1016/j.diagmicrobio.2008.09.016

28. Cairns L, Blythe D, Kao A, Pappagianis D, Kaufman L, Kobayashi J, et al. Outbreak of coccidioidomycosis in Washington state residents returning from Mexico. Clin Infect Dis. 2000;30:61–4. PubMed http://dx.doi.org/10.1086/313602

29. Centers for Disease Control and Prevention. Coccidioidomycosis in travelers returning from Mexico—Pennsylvania, 2000. MMWR Morb Mortal Wkly Rep. 2000;49:1004–6. PubMed

30. Wright PW, Pappagianis D, Wilson M, Louro A, Moser SA, Komatsu K, et al. Donor-related coccidioidomycosis in organ transplant recipients. Clin Infect Dis. 2003;37:1265–9. PubMed http://dx.doi.org/10.1086/378741

31. Clark TAHS, Johnson E, Mark K, Werner SB, Lindsley M, Evans B, et al. Coccidioidomycosis associated with the World Championship of Model Airplane Free Flight—Lost Hills, California, 2001. In: Proceedings of the Annual Coccidioidomycosis Study Group Meeting; 2002 Apr 6; Davis (CA). Mycopathologia. 2004:157:9.

32. Crum N, Lamb C, Utz G, Amundson D, Wallace M. Coccidioidomycosis outbreak among United States Navy SEALs training in a *Coccidioides immitis*–endemic area—Coalinga, California. J Infect Dis. 2002;186:865–8. PubMed http://dx.doi.org/10.1086/342409

33. Pappagianis D, Sacks HD. Outbreak of coccidioidomycosis in California prisons 2003–2004. In: Galgiani JN, Brauer RJ, editors. Proceedings of the Annual Coccidioidomycosis Study Group Meeting; 2004 Apr 3; Rosarito Beach, Mexico. Tucson (AZ); Valley Fever Center for Excellence; 2004. p. 6.

34. Cummings KC, McDowell A, Wheeler C, McNary J, Das R, Vugia DJ, et al. Point-source outbreak of coccidioidomycosis in construction workers. Epidemiol Infect. 2010;138:507–11. PubMed http://dx.doi.org/10.1017/S0950268809990999

35. Engelthaler DM, Chiller T, Schupp JA, Colvin J, Beckstrom-Sternberg SM, Driebe EM, et al. Next-generation sequencing of *Coccidioides immitis* isolated during cluster investigation. Emerg Infect Dis. 2011;17:227–32. PubMed http://dx.doi.org/10.3201/eid1702.100620

36. Brillhante RSN, Moreira Filho RE, Rocha MFG, Castelo-Branco DS, Fechine MAB, Lima RA, et al. Coccidioidomycosis in armadillo hunters from the state of Ceará, Brazil. Mem Inst Oswaldo Cruz. 2012;107:813–5. PubMed http://dx.doi.org/10.1590/S0074-02762012000600017

37. Wilken JA, Sondermeyer G, Shusterman D, McNary J, Vugia DJ, McDowell A, et al. Coccidioidomycosis among workers constructing solar power farms, California, USA, 2011–2014. Emerg Infect Dis. 2015;21:1997–2005. PubMed http://dx.doi.org/10.3201/eid2111.150129

38. Wilken JA, Marquez P, Terashita D, McNary J, Windham G, Materna B; Centers for Disease Control and Prevention (CDC). Coccidioidomycosis among cast and crew members at an outdoor television filming event—California, 2012. MMWR Morb Mortal Wkly Rep. 2014;63:321–4. PubMed
